# Normative Data of Dutch Idiomatic Expressions: Subjective Judgments You Can Bank on

**DOI:** 10.3389/fpsyg.2019.01075

**Published:** 2019-05-14

**Authors:** Ferdy Hubers, Catia Cucchiarini, Helmer Strik, Ton Dijkstra

**Affiliations:** ^1^Centre for Language Studies, Radboud University Nijmegen, Nijmegen, Netherlands; ^2^Centre for Language and Speech Technology, Radboud University Nijmegen, Nijmegen, Netherlands; ^3^Donders Institute for Brain, Cognition and Behaviour, Radboud University Nijmegen, Nijmegen, Netherlands

**Keywords:** idiomatic expressions, Dutch, subjective judgements, reliability, idiom knowledge

## Abstract

The processing of idiomatic expressions is a topical issue in empirical research. Various factors have been found to influence idiom processing, such as idiom familiarity and idiom transparency. Information on these variables is usually obtained through norming studies. Studies investigating the effect of various properties on idiom processing have led to ambiguous results. This may be due to the variability of operationalizations of the idiom properties across norming studies, which in turn may affect the reliability of the subjective judgements. However, not all studies that collected normative data on idiomatic expressions investigated their reliability, and studies that *did* address the reliability of subjective ratings used various measures and produced mixed results. In this study, we investigated the reliability of subjective judgements, the relation between subjective and objective idiom frequency, and the impact of these dimensions on the participants’ idiom knowledge by collecting normative data of five subjective idiom properties (Frequency of Exposure, Meaning Familiarity, Frequency of Usage, Transparency, and Imageability) from 390 native speakers and objective corpus frequency for 374 Dutch idiomatic expressions. For reliability, we compared measures calculated in previous studies, with the D-coefficient, a metric taken from Generalizability Theory. High reliability was found for all subjective dimensions. One reliability metric, Krippendorff’s alpha, generally produced lower values, while similar values were obtained for three other measures (Cronbach’s alpha, Intraclass Correlation Coefficient, and the D-coefficient). Advantages of the D-coefficient are that it can be applied to unbalanced research designs, and to estimate the minimum number of raters required to obtain reliable ratings. Slightly higher coefficients were observed for so-called experience-based dimensions (Frequency of Exposure, Meaning Familiarity, and Frequency of Usage) than for content-based dimensions (Transparency and Imageability). In addition, fewer raters were required to obtain reliable ratings for the experience-based dimensions. Subjective and objective frequency appeared to be poorly correlated, while all subjective idiom properties and objective frequency turned out to affect idiom knowledge. Meaning Familiarity, Subjective and Objective Frequency of Exposure, Frequency of Usage, and Transparency positively contributed to idiom knowledge, while a negative effect was found for Imageability. We discuss these relationships in more detail, and give methodological recommendations with respect to the procedures and the measure to calculate reliability.

## Introduction

There is a long-standing tradition of research investigating the processing of idiomatic expressions. Assuming that such expressions are stored as chunks with dedicated abstract representations, an analysis of their processing can teach us a lot about how sentence context interacts with the recognition of isolated target words. Unfortunately, large numbers of variables affect idiomatic processing, like familiarity, transparency, and imageability (Cacciari and Glucksberg, 1995; Cieślicka, 2006, 2013; Steinel et al., 2007; Libben and Titone, 2008; Carrol et al., 2017). Studies investigating idiom properties have led to ambiguous results. As an example, Libben and Titone (2008) conducted a series of behavioral experiments on idiom processing and examined the effect of idiom properties on response times. In one of the experiments they found a positive effect of transparency, whereas in another experiment this effect turned out to be absent. A study by Steinel et al. (2007) reported a positive effect of imageability, whereas Cacciari and Glucksberg (1995) found that imageability negatively affected idiom processing.

The equivocal results are not only due to the large number of variables and context-sensitivity. Data on idiom properties are usually obtained through norming studies in which subjective judgements are collected for various properties of idiomatic expressions. A consideration of various idiom norming studies shows that the operationalization of relevant variables differs across studies (Libben and Titone, 2008; Tabossi et al., 2011; Bonin et al., 2013; Nordmann et al., 2014; Beck and Weber, 2016a,b; Carrol et al., 2017; Nordmann and Jambazova, 2017). For example, in their norming study, Libben and Titone (2008) define familiarity as the extent to which participants have seen, heard or used the idiom, whereas Carrol et al. (2017) use familiarity to refer to the extent participants are familiar with the idiom. Obviously, we will only be able to make some progress in this research domain when relevant dimensions are identified and well-defined. In addition, it is of paramount importance for an appropriate interpretation of the collected data that the dimensions in question are measured reliably. Reliability is “the extent to which measuring instruments (raters) covary, i.e., give relative values which are correlated” (Rietveld and van Hout, 1993, p. 188). Moreover, reliability needs to be checked before subjective data in subsequent research can be trusted.

However, not all studies that presented normative data on idiomatic expressions investigated their reliability. Studies that did address reliability or agreement of subjective ratings (Titone and Connine, 1994; Tabossi et al., 2011; Bonin et al., 2013; Nordmann et al., 2014; Citron et al., 2016;Nordmann and Jambazova, 2017) employed a whole range of definitions of idiom properties and data collection methods and calculated different measures of reliability. As a result, some studies reported high reliability (Bonin et al., 2013; Citron et al., 2016), whereas others observed low reliability (Titone and Connine, 1994; Tabossi et al., 2011; Nordmann et al., 2014; Nordmann and Jambazova, 2017).

The goal of the present paper is threefold. First, we investigate the reliability of subjective judgements. To this end, we will obtain judgements of various properties of idiomatic expressions that have been prominent in the idiom literature (Frequency of Exposure, Frequency of Usage, Meaning Familiarity, Imageability, and Transparency), and propose the Dependability or D-coefficient (Brennan, 2001) as a measure of reliability, which is relatively unknown in this field of study. Second, we study the relationship between subjective ratings of frequency of exposure and objective ratings of frequency as obtained from corpora. Third, we include an objective measure of idiom knowledge based on meaning recognition for investigating how idiom properties characterized by reliable subjective ratings affect idiom knowledge.

To address these issues, the paper is organized as follows. First, we review previous studies investigating subjective idiom ratings, analyzing how they define the idiom properties under study and apply various reliability measures. Next, we describe how we collected subjective ratings of Dutch idioms for the properties mentioned above in a group of Dutch participants. The ratings are used to calculate different measures of reliability, including the Dependability or D-coefficient. We also assess to what extent reliably measured idiom properties are interrelated and how they affect participants’ knowledge of Dutch idioms. Finally, we discuss our results in relation to those of previous studies and give some methodological recommendations, proposing the D-coefficient for use in future studies.

### Properties of Idiomatic Expressions

The idiom properties familiarity, transparency, and imageability play a central role in the idiom literature and have been operationalized and defined differently across studies. Familiarity is known to influence idiom processing and is therefore often studied in this type of research. Familiarity has often been defined as “subjective frequency,” indicating how often a given word or idiom is encountered by a speaker (Gernsbacher, 1984; Titone and Connine, 1994; Beck and Weber, 2016a). According to some other authors familiarity “also incorporates how well a meaning is known or understood” (Nordmann et al., 2014, p. 88). Some authors have explicitly addressed this dimension of familiarity by asking subjects to what extent they know the idiom (Cieślicka, 2013) or to indicate how meaningful they find an expression to be (Tabossi et al., 2011). This has also been viewed as a measure of knowledge, albeit one based on subjective self-report.

On closer examination, the terms idiom frequency, familiarity, and knowledge can be taken to refer to distinct, but partially overlapping dimensions. With respect to frequency, a distinction can be drawn between subjective and objective frequency of exposure. The first one could refer to perceived frequency, or the intuition a speaker has of having come across a given expression, while objective frequency can refer to frequency as measured from corpora. However, collecting such objective frequency data for idiomatic expressions is not trivial. First, because it is not immediately clear from which corpus they should be collected, and we know that frequency data are going to vary depending on the corpus used (Gries and Ellis, 2015). Second, because owing to the flexible nature of idiomatic expressions, it can be challenging to collect objective idiom frequency from corpora.

Similarly, with respect to familiarity/knowledge, we can discern a subjective variant that indicates to what extent a speaker thinks (s)he is familiar with the meaning of the expression, and a more objective one that indicates to what extent a speaker really knows the expression (subjective and objective meaning familiarity). An additional dimension may be distinguished that refers to the extent to which speakers use the idiomatic expression themselves, i.e., self-reported frequency of usage. This dimension has not been included in previous studies, but it may be interesting to investigate in the framework of studies on idiom knowledge and idiom production. Therefore, it will be included in the present study (see below).

Imageability, defined as the extent to which a word, or an idiom for that matter, can be associated with a specific image, has been shown to facilitate learning (Paivio et al., 1966). This effect could be a consequence of an additional form of coding beyond verbal coding (Paivio, 1969, p. 257). However, studies on idiom imageability provide rather mixed results. Research on idiom acquisition has indeed shown that imageability has a positive effect on idiom learning (Steinel et al., 2007; Boers et al., 2008), but Cacciari and Glucksberg (1995) reported a negative effect of imageability on idiom processing. They found that participants more often depict the literal meaning than the figurative one. In addition, Carrol et al. (2017) did not find a clear effect of imageability. However, in their study imageability scores were not obtained from the subjects involved in the study, but were extrapolated from the concreteness norms for single words gathered by Brysbaert et al. (2014).

Transparency is an important property of idiomatic expressions that is often included in idiom processing studies. The exact definition of transparency, however, is not always made explicit and studies have been found to differ in this respect. A clear explanation is provided by Steinel et al. (2007), who refer to the distinction made by Geeraerts (1995) between *syntagmatic transparency* and *paradigmatic transparency*.

*Syntagmatic transparency* is defined as the “one-to-one correspondence between the formal structure of the expression and the structure of its semantic interpretation, in the sense that there exists a systematic correlation between parts of the semantic value of the expression as a whole and the constituent parts of that expression” (Geeraerts, 1995, p. 61). This definition of *syntagmatic transparency* comes close to what other authors refer to as *analyzability* (Gibbs and Nayak, 1989; Cacciari and Glucksberg, 1991; Glucksberg, 1993), *semantic decomposition* (Nunberg, 1979), or *semantic decomposability* “how the individual meanings of the idiom’s component words contribute to the figurative meaning of the phrase” (Titone et al., 2015, p. 173), or “the degree to which individual meanings of an idiom contribute to its overall figurative interpretation” (Cieślicka, 2015, p. 213).

*Paradigmatic transparency*, on the other hand, is defined as the “transparency of the semantic extension that leads from the original meaning of an expression to its transferred reading” (Geeraerts, 1995, p. 61). This corresponds to the definition of transparency adopted by Cieślicka (2015, p. 213): “The extent to which the original metaphorical motivation of an idiomatic phrase can be deduced from its literal analysis.” According to this author, the notions transparency and semantic decomposability have often been used interchangeably, while in fact they refer to distinct properties.

Carrol et al. (2017) refer to this distinction by Cieślicka (2015), but eventually opt for another operationalization of transparency and decomposability based on “the stage at which the judgment is being made.” In their study, transparency was operationalized as how easily subjects thought they could guess the meaning of the idiom based on the individual words, but without being shown the meaning. Decomposability was defined in the same way, but ratings were obtained later and by showing subjects the correct meaning of the idioms. In between these two questions subjects answered multiple-choice items aimed at testing their knowledge of meaning. While these answers gave the authors information about whether the subjects knew the meanings of the idioms, it is still unclear what the subjects were actually judging when they were asked to rate transparency. Because the actual meaning was not shown, they might have had a different meaning in mind than the correct one, even a meaning that was not included in the multiple-choice items. This complicates the interpretation of the changes in ratings from transparency to decomposability. Further discussion of the relationship between transparency and decomposability (Carrol et al., 2017, p. 17) does not clarify this point. In the current study, we define transparency as *paradigmatic transparency* (Geeraerts, 1995), which is also in line with the definition of Cieślicka (2015).

### Reliability of Subjective Judgements

The variability in various definitions of idiom properties makes it difficult to compare the results of different studies. Moreover, the operationalization of the variables may influence the reliability of the subjective judgements. When the definitions of the dimensions investigated are not unequivocal, participants may be induced to resort to their own interpretations, which in turn may affect the reliability of their ratings. To test this view, we teased apart these interrelated, but distinct dimensions, by proposing different evaluation scales with more precise definitions (see below, and Hubers et al., 2016; van Ginkel et al., 2016).

Other important elements that may further affect reliability are the research design and the sample size. Most studies collected data using a between-subject design (different groups of participants rated different dimensions of idiomatic expressions), because by using a within-subject design (all participants answered all questions), the ratings on one dimension may be influenced by the ratings on the other dimensions. However, Nordmann and Jambazova (2017) found no effects of study design (within-subjects vs. between-subjects) on idiom ratings. Moreover, “it is important to collect these ratings within subjects, because they can never be independent and should not be treated as such” (Nordmann and Jambazova, 2017, p. 200). In addition, they found that increasing the size of the sample did not improve reliability.

Studies that examined reliability of idiom ratings also differ from each other with respect to the measure of agreement and reliability adopted. This may have consequences for the interpretation of the results concerning reliability. Some studies calculated percentage agreement as a measure of reliability. Titone and Connine (1994), for example, collected normative data for 171 English idioms on various dimensions from groups of 28–30 native speakers of English. For familiarity and literality they employed 7-point scales, but did not measure reliability. In line with Gibbs et al. (1989), they treated decomposability as a categorical variable distinguishing three categories, and calculated percentage agreement. They concluded: “reliable agreement for compositionality was not found in our study.” Tabossi et al. (2011) gathered subjective judgements for 245 Italian idiom among 740 Italian native speakers. Groups of at least 40 subjects judged different lists of idioms on various properties on 7-point scales. Reliability was not measured for any of the scales. However, for the compositionality ratings, the percentages of agreement among subjects were compared to those of previous studies by Gibbs and Nayak (1989) and Titone and Connine (1994). In order to do this, the 7-point compositionality scale was converted to a binary variable (composable–decomposable). As observed by Nordmann et al. (2014), it is unclear what the advantage is of using a 7-point scale if the judgments are then treated as categorical, because in this case relevant information is lost. Tabossi et al. (2011) observed that “for most of the 245 idiomatic expressions judgments were not consistent,” and that “This inconsistency is disturbing as all the studies used the same procedure.” However, the use of percentage agreement is problematic, as this metric does not take chance agreement into account, which makes comparisons across studies difficult. Furthermore, the terms agreement and consistency are used interchangeably here and a measure of agreement for nominal variables, percentage agreement, is used for compositionality ratings on a 7-point scale for which the authors also compute mean and *SD* values (interval level of measurement).

Another metric that has been used in previous literature is Krippendorff’s alpha, an index that is suitable for variables at the nominal, ordinal, interval, and ratio level of measurement (Nordmann et al., 2014; Nordmann and Jambazova, 2017). Nordmann et al. (2014) gathered subjective judgments of various idiom properties through 7-point Likert scales from 44 native speakers and 32 non-native speakers of English for 100 English idioms. The reliability values obtained were quite low for both the native and the non-native judgments: They varied between -0.02 (familiarity judged by non-natives) and 0.27 (familiarity judged by natives). Nordmann and Jambazova (2017) describe two rating studies in which reliability (or agreement, the terms are used interchangeably) was measured. The first study employed a larger sample of 160 Bulgarian subjects who rated 90 Bulgarian idioms and a smaller group of 36 English subjects who rated English translations of the Bulgarian idioms. Idiom properties were rated on 7-point Likert scales. Again Krippendorff’s alpha was computed and the reliability values appeared to be low in this case too (between 0.124 for decomposability and 0.385 for literality) both for the larger and the smaller groups of subjects. In the second study 32 English native speakers were involved in a within-subject rating and 120 took part in between-subject ratings in which four groups of 30 participants rated the same idiom properties as in Study 1. Reliability was low across the board (between 0.217 for meaning and 0.332 for familiarity). Inspection of the Supplementary Materials provided with this paper shows that the authors calculated Krippendorff’s alpha for ordinal variables. It is not completely clear whether Likert scales should be treated as ordinal or interval variables, but it surprising to treat them as interval variables for computing mean and *SD* values and as ordinal variables for computing reliability.

The low reliability scores obtained in the studies discussed above may be due to the measures used. Both Krippendorff’s alpha and percentage agreement are measures of agreement instead of reliability (Tinsley and Brown, 2000). Agreement concerns the absolute values of a set of ratings, and indicates to what extent the values are identical. Reliability, on the other hand, indicates to what extent a set of ratings covary. Reliability can be high even if the absolute values are not identical. Because reliability is based on measures of covariation and correlation, “reliability analysis requires an interval level of measurement” (Rietveld and van Hout, 1993, p. 188). So, the use of agreement or reliability metrics is related to the level of measurement of the variables involved: nominal and ordinal for agreement, and interval for reliability (Rietveld and van Hout, 1993; de Vet et al., 2006). Moreover, as Rietveld and van Hout (1993) further explain, reliability and agreement measure different aspects of a set of ratings. This point is best illustrated by the discussion presented in Nordmann et al. (2014, p. 93) when they present an analogy from essay assessment: Two teachers assign different grades to two essays by the same student, and the grades by the two teachers for each essay are not identical, but they are strongly correlated. This is a typical case in which a reliability measure will return a high value, but an agreement measure a low one. Nordmann et al. (2014, p. 93) suggest that in the case of normative judgments of idiom properties, we are interested in covariation between the raters and correlation between the ratings, and not so much in whether the values of the ratings are identical. It follows that in these cases we should compute measures of reliability, not of agreement.

Another metric that has been used in previous research and that does measure reliability is the Intraclass Correlation Coefficient (ICC) (Shrout and Fleiss, 1979). Bonin et al. (2013) collected normative data for 305 French idioms from groups of 23–30 French native speakers through 5-point scales. The ICC with random effects of both participants and items was used to measure reliability, obtaining values between 0.81 for age of acquisition and 0.96 for subjective frequency. The ICC is an appropriate reliability measure for interval variables, and the parameter setting with random effects of both participants and items allows a generalization to raters not included in the sample. Citron et al. (2016) employed 7-point scales to collect subjective judgements of various idiom properties from 624 German idiomatic expressions by 249 native speakers. Reliability was measured through Cronbach’s alpha, a particular case of the ICC, obtaining values between 0.80 for familiarity and 0.98 for emotional valence. For Cronbach’s alpha raters are treated as a fixed factor and items as random. This parameter setting produces the highest values of ICC. The downside is that in this case the results cannot be generalized to raters not included in the sample (see for further details Rietveld and van Hout, 1993).

The ICC with random effects of both participants and items seems to be the most appropriate reliability measure. It calculates reliability, not agreement, and it allows to generalize across raters. However, the choice of a reliability coefficient may also depend on the presence of missing values. The ICC requires a fully crossed design in which all participants rate all items and is unable to handle missing values. Ideally, we would like to apply a coefficient that can take all these factors into account so as to allow comparisons between studies that differ in various respects from each other. The Dependability coefficient (D-coefficient) based on Generalizability Theory (Brennan, 2001) is one such coefficient.

Generalizability Theory is a statistical theory for evaluating the reliability of behavioral measurements, such as object ratings (Shavelson and Webb, 1991, 2006; Brennan, 2001). The metric proposed for measuring reliability in this framework, and that seems particularly suited for subjective ratings of idiom properties, is the Dependability or D-coefficient. This metric, based on the ICC, takes into account the estimated variance in items and raters, and is also able to account for the variance in other fixed and random factors (Brennan, 2001; Rietveld and van Hout, unpublished). The D-coefficient has considerable advantages, the most important being that it can take into account sources of variance other than items and raters, and that it can handle different research designs. Regarding the latter, in addition to the fully crossed designs in which each rater judges each item (needed to calculate other reliability measures), Generalizability Theory also allows for unbalanced research designs, in which different groups of participants rate different groups of objects (Brennan, 2001). Another advantage of this statistical theory is that it allows to easily calculate the minimum number of raters required to obtain reliable data (Shavelson and Webb, 2006; Li et al., 2015). Based on the collected ratings, the number of raters, but also the number of items, can be manipulated to see what the consequences would be for the reliability of the data.

### The Present Study

Our literature review indicates that research on the reliability of subjective judgments of idiom properties so far has been limited and has produced mixed results. Analyses of the studies that investigated reliability reveal a variety of procedures and metrics and suggest that the discrepancies in results may be due to the methods and metrics employed. In the present study, we focused on such reliability issues. In addition, for the idiom properties that could be reliably measured, we investigated their relation with objective idiom knowledge. To that end, we collected and analyzed subjective judgments of frequency of exposure, meaning familiarity, frequency of usage, imageability and transparency of Dutch idiomatic expressions by Dutch native speakers and their scores on a test of objective knowledge of idiom meaning.

Next, we formulated three research questions. First, we wished to know how reliable subjective judgements of various idiom properties actually are. Thus, we computed their reliability in newly collected data using Generalizability Theory. In line with Bonin et al. (2013), we expected high reliability values for our ratings, combining the suitability of this technique with more precise definitions and operationalizations of relevant idiom properties. In addition, subjective ratings of frequency of exposure, frequency of usage, and meaning familiarity were expected to be more reliable than ratings of imageability and transparency, because research indicates that these latter two dimensions are generally more difficult to assess than frequency of exposure, and meaning familiarity. This increased difficulty may be due to a difference in the relation to the idiom. The dimensions meaning familiarity, frequency of exposure, and frequency of usage reflect the native speakers’ experience with idiomatic expressions. Because formulaic language, which idiomatic expressions are part of, is found to be generally known by native speakers (Pollio et al., 1977; Erman and Warren, 2000; Wray and Perkins, 2000), and their experience with idiomatic expressions is rather comparable, we expected judgements of these experience-based dimensions to show relatively little variation. The dimensions transparency and imageability, which are more closely related to the content words of the idiomatic expressions, are expected to show more variation. Consequently, subjective judgments of content-related dimensions are expected to be less reliable than judgments of experience-based dimensions.

Second, we wondered to what extent subjective idiom frequency, as assessed in our study, is related to objective idiom frequency as measured from corpora. While subjective and objective frequency have been compared for single words and collocations (Siyanova-Chanturia and Spina, 2015), to our knowledge such systematic comparisons have not been conducted for idiomatic expressions. For single words subjective and objective frequencies appeared to be strongly correlated, whereas for collocations a more complex picture emerged (Siyanova-Chanturia and Spina, 2015). Subjective frequency intuitions of high frequency collocations correlated strongly with objective frequency, as taken from corpora. For medium and low frequency collocations the subjective frequency judgements and objective frequency correlated poorly. As mentioned above, collecting objective frequency data for idiomatic expressions is difficult for a number of reasons related to the choice of the corpus from which the data should be obtained and the flexible nature of idiomatic expressions. We decided to collect this information from the SoNaR corpus (Oostdijk et al., 2013), a large corpus of written Dutch. Previous research has shown that subjective frequency of idiomatic expressions is generally relatively high in native speakers (e.g., Bonin et al., 2013; Beck and Weber, 2016b). As to objective frequency, there are indications that while idiomatic expressions as a general phenomenon are frequent, individual idioms are rather infrequent (Ellis, 2012). Based on these findings we expect correlations between subjective and objective idiom frequency to be low.

Third, we were interested to know how different subjective idiom properties and objective idiom frequency are in fact related to objectively assessed idiom knowledge. To answer this question, we reviewed the psycholinguistic literature. Many studies on idiom processing investigated the role of idiom properties in processing (e.g., Cacciari and Tabossi, 1988; Gibbs et al., 1989; Libben and Titone, 2008; Cieślicka, 2013; Titone and Libben, 2014). Only two studies, however, sought to identify idiom properties that are important predictors of offline comprehension measures such as idiom knowledge and subjective familiarity.

Carrol et al. (2017) examined the role of familiarity, and transparency in correctly identifying the meaning of English idiomatic expressions in a multiple-choice question. Familiarity was operationalized as the extent to which participants were familiar with the idiom. Transparency was operationalized as the extent to which participants were able to guess the meaning of the phrase based on the individual words. Carrol et al. (2017) found that Familiarity was a good predictor of objective idiom knowledge, whereas transparency was not found to contribute to idiom knowledge.

Libben and Titone (2008) investigated the impact of idiom properties on the meaningfulness of English idiomatic expressions. Meaningfulness, operationalized as the extent to which participants considered the phrase to be meaningful, can be seen as an indirect and subjective measure of idiom knowledge. In a regression analysis on the aggregated data, the authors examined to what extent familiarity, semantic decomposability, literal plausibility, noun frequency, and verb frequency influenced the meaningfulness ratings. Familiarity was operationalized as what we would define as frequency: the extent to which the participant has seen, heard or used the idiom. In line with our terminology, we use the term frequency of exposure instead. Frequency of exposure turned out to be an important predictor. The more frequent an idiomatic expression in daily life, the more familiar participants judged this expression to be. Semantic decomposability turned out to be important for infrequent idiomatic expressions only. If an infrequent idiomatic expression was semantically decomposable, people indicated to be more familiar with the idiom, as compared to if the idiom was semantically non-decomposable. The other factors included in the analysis did not significantly influence meaningfulness ratings of English idiomatic expressions.

Both reviewed studies investigated the impact of idiom properties on idiom knowledge. However, Carrol et al. (2017) only examined the effect of familiarity and transparency on idiom knowledge and the operationalization of familiarity was imprecise. Participants could have assessed familiarity with respect to idiom meaning or form. This makes it difficult to interpret the observed positive effect of familiarity on idiom knowledge. Libben and Titone (2008) did investigate the effect of more idiom properties on idiom knowledge, but they assessed idiom knowledge indirectly and subjectively. This assessment shows whether people think they know the meaning of an idiomatic expression, but does not directly tap into the participant’s actual idiom knowledge. To investigate how idiom properties influence idiom knowledge, this should be assessed objectively, allowing comparisons between offline (rating) and online comprehension (reaction time) data.

In our study, we investigated the effect of subjective idiom properties and objective idiom frequency on idiom knowledge in more detail. We obtained objective frequency data from a large corpus of written Dutch (Oostdijk et al., 2013) and assessed objective idiom knowledge through multiple-choice questions about the meaning of Dutch idiomatic expressions. We examined more subjective idiom properties than in Carrol et al. (2017), and distinguished three operationalizations of general familiarity: subjective meaning familiarity, subjective frequency of exposure, and subjective frequency of usage. Subjective familiarity is associated with the meaning of the idiom. Subjective frequency is defined as the idiom’s occurrence in daily life (familiarity with the form), and Subjective usage is the extent to which participants indicate to actively use the idiomatic expression themselves. For readability’s sake we try to limit the use of the term subjective and opt for the labels Familiarity and Usage, but we maintain Subjective Frequency as opposed to Objective Frequency. We also included Transparency and Imageability in our analysis as predictors of Objective Idiom Knowledge.

Finally, we explored whether the measurements obtained through more precise operationalizations of general familiarity each uniquely contribute to objective idiom knowledge, and how they interact with other idiom properties, such as Transparency and Imageability. Based on the literature, we expected Familiarity, Frequency, and Transparency to have a positive effect on Objective Idiom Knowledge (Libben and Titone, 2008; Carrol et al., 2017). As to the effect of Imageability, previous research has been inconclusive. Earlier studies found positive effect of Imageability on idiom learning (Steinel et al., 2007; Boers et al., 2008), whereas it was found to negatively affect idiom processing (Cacciari and Glucksberg, 1995). Objective idiom frequency has not been studied before in this connection. However, other research findings lead to us to assume that objective frequency should have a positive effect on Objective Idiom Knowledge, albeit a less strong one than Subjective Frequency given that the latter is based on individual experience of the same participant.

## Materials and Methods

### Participants

In total, 390 native speakers of Dutch, mainly university students, participated in the rating study (350 female participants and 40 males). Their age varied between 18 and 30 (mean = 20.4; *SD* = 1.5) and about 98% of them were highly educated.

This study was ethically assessed and approved by the Ethics Assessment Committee (EAC) of the Faculty of Arts of Radboud University Nijmegen (number 3382).

### Materials

We selected 374 Dutch idiomatic expressions and their appropriate meaning based on Dutch dictionaries (e.g., Stoett, 1925; Den Boon and Hendrickx, 2017; Slot Webcommerce, 2017), online idiom lists (Genootschap OnzeTaal, 2017), and our own knowledge and experience. We adjusted these meanings in such a way that they did not contain other idiomatic expressions. For example, to explain the Dutch expression *ergens mee voor de draad komen*, which means “to finally say something,” the dictionary uses another idiom *ergens mee voor de dag komen*. This expressions conveys the same meaning as the expression *ergens mee voor de draad komen*. Therefore, we formulated the meaning in another way without using an idiomatic expression: *iets vertellen* (“to tell something”). The database with the idiomatic expressions and the aggregated results is available in a repository^1^.

#### Objective Idiom Frequency

We collected objective idiom frequency information from the SoNaR corpus of written Dutch (Oostdijk et al., 2013), consisting of 500 million words. First, we identified one content word per idiom (usually a noun) and extracted all sentences from the corpus containing this content word. For example, we looked for all sentences containing the Dutch word *lamp* “lamp” in the corpus (from the Dutch idiom *tegen de lamp lopen* “to get caught”). Second, we obtained the sentences containing the idiomatic expressions in the subset by means of pattern matching, taking into account different word orders and inflections of the verb.

### Design and Procedure

#### Operationalization of Variables

Five subjective properties of idioms were rated on 5-point Likert scales: Subjective Frequency, Subjective Usage, Subjective Familiarity, Subjective Imageability, and Subjective Transparency (in the remainder of the paper these properties are referred to as Subjective Frequency, Usage, Familiarity, Imageability, and Transparency, respectively). Subjective Frequency is defined as the relative degree to which a participant indicates to have come across an idiomatic expression in speech or in print (Gernsbacher, 1984; Titone and Connine, 1994). Usage is defined as the frequency with which a subject indicates to have used an idiomatic expression. Familiarity is here conceived of as how well a speaker says to know the meaning of an idiom (Nordmann et al., 2014, p. 88). In line with Steinel et al. (2007), and Boers et al. (2008), Imageability is defined as the extent to which an idiom can evoke an image. Transparency is interpreted in line with (Cieślicka, 2015, p. 213) and paradigmatic transparency (Geeraerts, 1995, p. 61), i.e., the degree to which the semantic value of the entire expression can be understood in terms of the semantic values of its constituting words (Steinel et al., 2007). We also measured knowledge of idiom meaning through an objective multiple-choice test.

#### Questionnaire

The rating study was conducted online through the Qualtrics platform (Qualtrics, 2005). The participants filled in a background questionnaire with questions about gender, year of birth, place of residence, mother tongue, level of education, and language background. In the rating study, the participants answered five questions about the idiomatic expressions on 5-point Likert scales (questions 1, 2, 3, 4, and 7), one open question (question 5) and one multiple-choice item (question 6).

(1) Subjective Frequency*:* How often have you heard or read this expression? (1. very rarely – 5. very often).
(2) Usage: How often have you used this expression yourself? (1. very rarely – 5. very often).
(3) Familiarity*:* How familiar are you with the meaning of this expression? (1. completely unfamiliar – 5. completely familiar).
(4) Imageability: How easily can you form an image of this expression? (1. very hard – 5. very easily).
(5) Objective Idiom Knowledge (recall): What does this idiomatic expression mean? (open question, not further analyzed in this study).
(6) Objective Idiom Knowledge (recognition): Which definition is the correct one? (multiple-choice question: 4 alternatives).
(7) Transparency*:* How clear is the meaning of this expression based on the individual words in the expression? (1. very unclear – 5. very clear).

Since Nordmann and Jambazova (2017) did not find any effects of study design (within-subjects vs. between-subjects) on idiom ratings, we adopted a within-subject design in which all participants answered all questions. This way we take into account the relations between the idiom properties within the individual.

The idiomatic expressions were randomly divided over 15 experimental lists consisting of 25 idiomatic expressions. Every idiomatic expression occurred in only one list. Each participant rated one list of 25 idiomatic expressions and before doing this they rated 2 idiomatic expressions in a practice session in which the questions and the labels of the extreme points of the Likert scales were explained. As a form of calibration, examples were provided of idiomatic expressions representing the extreme values. On average, the participants completed the rating study in 30 min.

### Data Analysis

We calculated the mean ratings and standard deviations for all dimensions of each Dutch idiomatic expression. The average Objective idiom knowledge and its standard deviation were calculated based on the proportions correct on the multiple-choice question. To obtain a general overview of the data, we computed the correlations of these dimensions based on the individual data.

To gain insight into the potential differences between reliability measures employed in previous research, we calculated Krippendorff’s alpha, Cronbach’s alpha, and the ICC for the data on the different idiom properties obtained in the different experimental lists in our study. These measures were calculated using the “rel” package (Lo Martire, 2017) in R version 3.4.0 (R Development Core Team, 2008), and were averaged across lists. We also computed the Dependability coefficient using the “gtheory” package (Moore, 2016), both averaged across lists and based on the dataset as a whole. The ICC was calculated for the mean ratings with the parameters “two-way”, and “absolute agreement”, indicating random effects for participants and items. We refer to this specific instance of the ICC as *ICC(2,k)* (Shrout and Fleiss, 1979). To answer the research question on reliability, we compared the D-coefficients based on the dataset as a whole of the different idiom properties, and we calculated the minimum number of raters required to obtain reliable data.

Based on the outcomes of the reliability analyses, we performed logistic mixed effects regression analyses to answer our second research question about the contribution of the different subjective idiom properties to Objective idiom knowledge. These analyses were conducted in the statistical software package “R” version 3.4.0 (R Development Core Team, 2008), and the R packages “lme4” (Bates et al., 2015), “lmerTest” (Kuznetsova et al., 2017), and “effects” (Fox, 2003) were used. The models were built in a forward manner, starting off with a basic model including a random intercept for participants and fixed effects of the idiom properties under study. Subsequently, we added different predictors (random and fixed factors) one by one to the model based on theory, and examined whether the model fit improved. If this was not the case, we decided not to include this predictor in the model. The final model is reported in this paper.

## Results

### General Results

Table 1 presents a summary of the ratings. In general, participants seem to be exposed to idiomatic expressions quite frequently (mean = 3.41; *SD* = 1.39), and use idiomatic expressions to a lesser extent (mean = 2.17; *SD* = 1.30). Idiom knowledge is quite high (85.48% correct). See Supplementary Figure S1 for the distribution of the individual ratings for the idiom properties.

**Table 1:** Mean and SD for ratings on idiom properties and for performance on knowledge question.

Idiom property	Mean (*SD*)
Subjective Frequency (scale 1–5)	3.41 (1.39)
Familiarity (scale 1–5)	3.08 (1.35)
Usage (scale 1–5)	2.17 (1.30)
Transparency (scale 1–5)	3.08 (1.28)
Imageability (scale 1–5)	3.36 (1.33)
Objective idiom knowledge (in %)	85.48 (35.22)

Pearson’s correlations were computed between the individual ratings for each idiom on all rating dimensions and the objective measures of idiom frequency and idiom knowledge (presented in Table 2). All subjective idiom properties significantly correlated with each other, with high values for Subjective Frequency, Familiarity, and Usage (Pearson’s *r* > 0.65). Transparency showed the highest correlation with Objective Idiom Knowledge (Pearson’s *r* = 0.35). Objective Frequency correlated relatively poorly with the subjective idiom frequency judgements (Pearson’s *r* = 0.20), the other subjective judgment scales (Pearson’s *r* < 0.19), and with Objective Idiom Knowledge (Pearson’s *r* = 0.08).

**Table 2:** Correlation matrix based on individual ratings of Dutch idiomatic expressions.

	Subjective					Objective idiom
	Frequency	Familiarity	Usage	Transparency	Imageability	knowledge
Familiarity	0.79^∗^					
Usage	0.66^∗^	0.68^∗^				
Transparency	0.28^∗^	0.32^∗^	0.29^∗^			
Imageability	0.35^∗^	0.38^∗^	0.27^∗^	0.34^∗^		
Objective idiom knowledge	0.30^∗^	0.33^∗^	0.24^∗^	0.35^∗^	0.13^∗^	
Objective Frequency	0.20^∗^	0.19^∗^	0.19^∗^	-0.02	-0.02	0.08^∗^

^∗^*p < 0.001*.

### Reliability

#### Reliability Measures per List

We computed the reliability measures for each list separately. Table 3 shows the reliability coefficients averaged over the lists. Both the D-coefficient, Cronbach’s alpha, and the ICC(2,k) reflect high reliability for each of the idiom properties (all coefficients >0.85). The D-coefficient and the ICC(2,k) are identical, and Cronbach’s alpha is somewhat higher. However, the reliability as reflected by Krippendorff’s alpha is much lower for all properties (all coefficients <0.41). The ratings on Subjective Frequency, Familiarity and Usage seem to be more reliable than the Transparency and Imageability ratings, as indicated by all reliability measures. For a full overview of the coefficients per list see Supplementary Table S1.

**Table 3:** Mean reliability coefficients, SDs, and range for each idiom property averaged over lists.

	D-coefficient		ICC(2,k)		Cronbach’s alpha		Krippendorff’s alpha	
	Mean (*SD*)	Min–max	Mean (*SD*)	Min–max	Mean (*SD*)	Min-max	Mean (*SD*)	Min–max
Subjective Frequency	0.943 (0.023)	0.894–0.975	0.943 (0.023)	0.894–0.975	0.957 (0.015)	0.924–0.979	0.402 (0.090)	0.281–0.553
Familiarity	0.943 (0.024)	0.909–0.973	0.943 (0.024)	0.909–0.973	0.958 (0.016)	0.928–0.979	0.403 (0.096)	0.283–0.558
Usage	0.932 (0.033)	0.865–0.966	0.932 (0.033)	0.865–0.966	0.976 (0.020)	0.914–0.976	0.365 (0.089)	0.201–0.470
Transparency	0.866 (0.043)	0.771–0.912	0.866 (0.043)	0.771–0.912	0.905 (0.032)	0.834–0.947	0.201 (0.060)	0.109–0.305
Imageability	0.877 (0.056)	0.738–0.934	0.877 (0.056)	0.738–0.934	0.906 (0.038)	0.820–0.948	0.228 (0.076)	0.092–0.389

#### Reliability Measures on Entire Dataset

Table 4 shows the D-coefficient for the different idiom properties calculated on the entire dataset, taking into account the nested design. The coefficients based on the full dataset are very similar to the averaged D-coefficients and ICCs presented in Table 3. The ratings for each of the idiom properties are highly reliable, but those for Subjective Frequency, Familiarity, and Usage are more reliable than those for Transparency and Imageability.

**Table 4:** D-coefficient for each idiom property based on the full dataset.

Idiom property	D-coefficient
Subjective Frequency	0.947
Familiarity	0.946
Usage	0.937
Transparency	0.872
Imageability	0.888

#### Reliability as a Function of the Number of Raters

**FIGURE 1:**
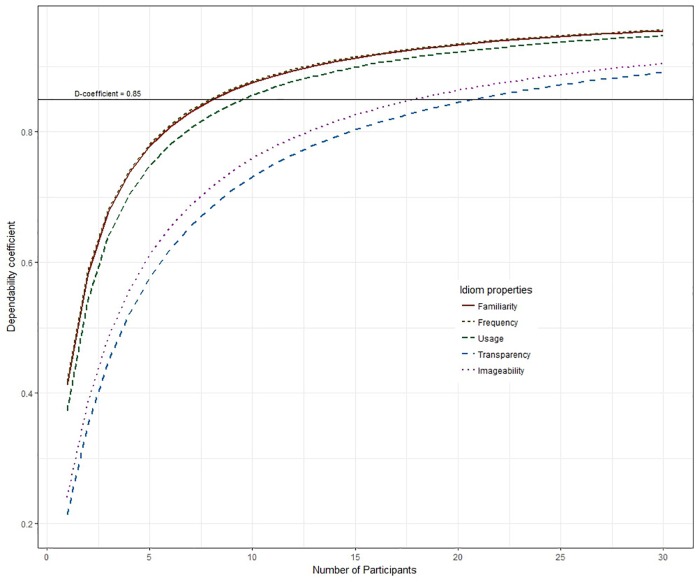
Dependability coefficient for a given number of raters.

The advantage of Generalizability Theory is that a reliability coefficient can be computed for every number of ratings based on the variance components estimated on the basis of the current data. Figure 1 shows the increase in reliability as a function of the number of participants in the rating study. The idiom properties Familiarity, Subjective Frequency, and Usage seem to require fewer raters to collect reliable data as compared to Transparency and Imageability. To obtain highly reliable ratings (D-coefficient >0.85) for Familiarity, Frequency, and Usage approximately 10 participants should be recruited. For Imageability and Transparency about 20 people are needed to obtain equally reliable data.

### Impact of Idiom Properties on Objective Idiom Knowledge

To examine which factors influence the participants’ knowledge of idiomatic expressions separately and in combination, we conducted a logistic mixed effects regression analysis. The multiple-choice question on idiom knowledge was converted into a binary variable expressing whether the multiple-choice question was answered correctly or not. This binary variable was used as the dependent variable in the regression analysis.

In our final model we included the following predictors as fixed effects: (1) Familiarity, (2) Transparency, (3) Imageability, (4) Subjective Frequency, (5) Usage, (6) Objective Frequency, and the interactions (7) Familiarity × Transparency, and (8) Familiarity × Transparency. All predictors were centered to account for multicollinearity, and Objective Frequency was log-transformed.

In addition, we included Idioms (random intercept only), and Participants as a random effect (random intercept and random slope of Imageability). The model is presented in Table 5. The variables included in the model were not collinear (see Variance Inflation Factors in Supplementary Table S2).

**Table 5:** Regression model with answer correct as the dependent variable.

Fixed effects	Estimate	Std. Error	*z* value	
(Intercept)	2.97484	0.19790	15.032	^∗∗∗^
Familiarity	0.45390	0.06469	7.016	^∗∗∗^
Transparency	0.88302	0.05114	17.268	^∗∗∗^
Imageability	-0.22449	0.04964	-4.522	^∗∗∗^
Subjective Frequency	0.13631	0.05165	2.639	^∗∗^
Usage	0.15305	0.06329	2.418	^∗^
Objective Frequency	0.16227	0.06099	2.660	^∗∗^
Familiarity × Transparency	-0.07417	0.02945	-2.518	^∗^
Familiarity × Imageability	-0.06873	0.02749	-2.500	^∗^

^∗^*p < 0.05, ^∗∗^*p* < 0.01; ^∗∗∗^*p* < 0.001*.

**FIGURE 2:**
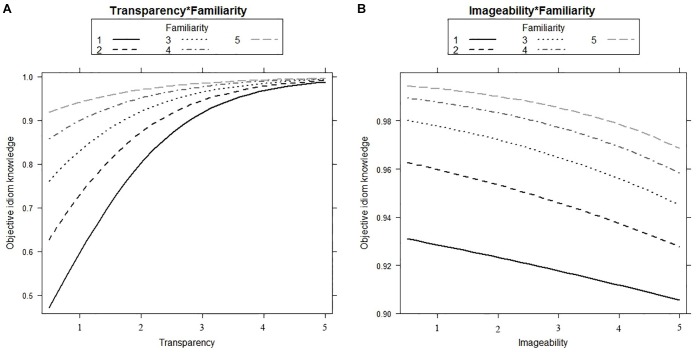
The effect of Transparency **(A)** and Imageability **(B)** on idiom knowledge for idiomatic expressions with different Familiarity ratings. The variables Transparency, Imageability, and Familiarity are rated on a scale from 1 (completely not transparent, imageable, and familiar respectively) to 5 (very transparent, imageable, and familiar respectively).

Familiarity has a positive effect on idiom knowledge (β = 0.45, *SE* = 0.07, *p* < 0.001). We also observed a positive effect of Transparency (β = 0.88, *SE* = 0.05, *p* < 0.001), Subjective Frequency (β = 0.14, *SE* = 0.05, *p* < 0.01), Usage (β = 0.15, *SE* = 0.06, *p* < 0.05), and Objective Frequency (β = 0.16, *SE* = 0.06, *p* < 0.01) on idiom knowledge. Furthermore, we found a negative effect of Imageability (β = -0.23, *SE* = 0.05, *p* < 0.001). The better people are able to form an image of the idiomatic expression, the worse their performance on the multiple-choice question. In addition, we observed a significant interaction of Familiarity and Transparency (β = -0.07, *SE* = 0.03, *p* < 0.05; see the left panel in Figure 2). The effect of Transparency on idiom knowledge is larger for idiomatic expressions that are not so familiar as compared to idiomatic expressions that are judged to be highly familiar. This is indicated by the steeper line for unfamiliar idioms than for familiar idioms. Familiarity and Imageability also significantly interact (β = -0.07, *SE* = 0.03, *p* < 0.05; see right panel of Figure 2). The more familiar participants are with the meaning of the idiomatic expression, the larger the negative effect of Imageability on idiom knowledge. This is indicated by the steeper lines for the familiar idioms than for unfamiliar idioms in the right panel of Figure 2.

## Discussion

This study is the first to provide subjective ratings on various dimensions of Dutch idiomatic expressions by native speakers. In order to increase the chances of obtaining an informative picture and reliable ratings, we adopted a more detailed operationalization of familiarity than was employed in previous studies. We found that native speakers indicate to be quite familiar with the meaning of idiomatic expressions, to frequently encounter idiomatic expressions in daily life, but to use them less often than that they encounter them. On average, the participants in our study judged the idiomatic expressions to be transparent, and apparently managed to easily form an image of these idiomatic expressions. The objective test of idiom recognition revealed that in general the idiom meanings are well-known. In addition, all subjective idiom properties positively correlated with each other and with Objective Idiom Knowledge.

Despite the difficulties in comparing results across studies because of different operationalizations of the same variables, our findings are in line with those obtained in norming studies on languages other than Dutch. High native speaker ratings of Frequency, and Familiarity are also found for English (Nordmann et al., 2014; Nordmann and Jambazova, 2017), French (Bonin et al., 2013), German (Citron et al., 2016), Italian (Tabossi et al., 2011), and Bulgarian (Nordmann and Jambazova, 2017). Transparency scores are quite comparable to those obtained in other studies (Bonin et al., 2013; Citron et al., 2016; Carrol et al., 2017).

### Reliability of Subjective Idiom Properties

Many studies collected normative data on idiomatic expressions and used these as a basis for psycholinguistic research. However, the majority of these studies did not examine whether the collected subjective ratings were in fact reliable (e.g., Libben and Titone, 2008; Carrol et al., 2017). Norming studies that calculated reliability used different metrics and obtained mixed results. Some studies reported poor reliability (Nordmann et al., 2014; Nordmann and Jambazova, 2017), whereas others found high reliability (Bonin et al., 2013; Citron et al., 2016). In this study we investigated the reliability of judgments of Dutch idiomatic expressions in more detail. We tried to operationalize our variables more precisely than in previous studies, which was expected to increase reliability. Furthermore, to decide which metric to use to assess reliability, we took into account specific aspects of the research design and the level of measurement of the variables. This led us to propose a metric that can be calculated based on the whole dataset and that is relatively unknown in this field of study, the D-coefficient. In contrast to the metrics used in previous studies, this measure can handle unbalanced research designs and missing data. By using this metric, we were also able to assess the minimum number of raters per dimension that are required to obtain reliable data. To show how adopting a metric that is less suitable for the research design can affect reliability, we also calculated the different metrics used in previous studies for our newly collected data.

We calculated different metrics for the individual lists of idiomatic expressions and found that almost all metrics reflect high reliability, except Krippendorff’s alpha. We obtained identical values for the ICC(2,k) and the D-coefficient, and slightly higher values (for all dimensions) for Cronbach’s alpha. The results of the metrics calculated based on the individual experimental lists show that it is important to use the appropriate metric. As we mentioned above, the ICC(2,k) or the D-coefficient should preferably be used. Krippendorff’s alpha is rather a measure of agreement than of reliability, which explains the lower values. Finally, Cronbach’s alpha does not consider raters as a random factor, which results in higher reliability scores.

The D-coefficients based on the dataset as a whole, were very similar to the D-coefficients averaged across lists. This shows that the lists were carefully constructed and that the factor List explains only a limited amount of variance. This does not mean, however, that we do not have to take into account the variance of the lists, because it could have been an important source of variance. Moreover, the idiom properties Familiarity, Subjective Frequency, and Usage are highly reliable. The reliability coefficients of the idiom properties Transparency and Imageability are slightly lower, although still very high. These results indicate that by precisely operationalizing the dimensions, using appropriate procedures to obtain the measurements, and by using the appropriate reliability metric, high reliability can be obtained for subjective judgements of idiomatic expressions.

Based on the reliability coefficients, the dimensions can be divided into two groups: the content-related dimensions Transparency and Imageability on the one hand, and the experience-based dimensions Subjective Frequency, Familiarity, and Usage on the other. This division becomes even more apparent if we consider the minimum number of raters that are required to obtain a reliability of 0.85. For Familiarity, Subjective Frequency, and Usage approximately 10 participants should be recruited to achieve a reliability of 0.85, whereas for Transparency and Imageability about 20 participants are needed. In line with our expectations, judging Transparency and Imageability seems to be more difficult than judging Familiarity, Subjective Frequency, and Usage. The experience-based dimensions Familiarity, Subjective Frequency, and Usage appear to be less susceptible to variation than the content-based dimensions Transparency and Imageability.

### Comparison Between Subjective and Objective Idiom Frequency

To gain more insight into the dimension frequency of exposure, we investigated the relation between subjectively assessed idiom frequency and objective idiom frequency as collected from a large corpus of written Dutch. In line with findings that idiomatic expressions are relatively infrequent (Ellis, 2012) and our expectations about the correlation, we found that Subjective Frequency indeed correlated relatively poorly with Objective Frequency.

As Siyanova-Chanturia and Spina (2015) suggested with respect to collocations, this may be due to the poor ability of people to judge frequency of exposure for low frequency items. An advantage of our study is that we could also check how Subjective and Objective Frequency relate to idiom knowledge. We did find a high correlation between Subjective Frequency and Objective Idiom Knowledge, whereas the correlation between Objective Frequency and Objective Idiom Knowledge was very low. This suggests that Subjective and Objective Frequency reflect different aspects of idiom frequency. Subjective Frequency as operationalized in our study is closer to individual experience and, apparently, is a better reflection of idiom knowledge than Objective Frequency as obtained from a large corpus of written Dutch. This is not surprising, since the subjective frequency judgements are collected from the same group of participants as the information on idiom knowledge.

In addition, significant correlations were observed between Subjective Frequency and both Imageability and Transparency, while these idiom properties were not related to Objective Frequency. In line with our argumentation and as suggested by one of our Reviewers, this could also explain why Subjective Frequency correlated more strongly with Objective Idiom Knowledge than Objective Frequency: Apparently Objective Frequency is unrelated to the idiom properties that improve idiom knowledge on their own (e.g., Transparency and Imageability). A more detailed study of objective and subjective idiom frequency, their development in native and non-native speakers, and their impact on idiom knowledge and idiom processing would constitute interesting topics for future research.

### Relation of Idiom Properties to Objective Idiom Knowledge

In order to gain more insight into how idiom properties influence idiom knowledge, we investigated how the different subjective idiom properties and Objective Frequency contribute to Objective Idiom Knowledge. We found that all idiom properties significantly impact idiom knowledge. We broke down general familiarity into three more precise operationalizations (Familiarity, Subjective Frequency, and Usage) to see whether each of them uniquely contributed to Objective Idiom Knowledge and how they interacted with other idiom properties. We expected most idiom properties to positively contribute to objectively assessed idiom knowledge. For Imageability, we did not have strong expectations, due to mixed results in earlier studies.

Familiarity, Subjective Frequency, and Usage were found to have a positive effect on Objective Idiom Knowledge, indicating that the more experience users have with the idiom (experience with the meaning, the form, and with using the idiom), the better their idiom knowledge. Although these dimensions are strongly correlated, there are no signs of multicollinearity in the regression analysis. This, in combination with the fact that all three predictors turn out to be significant in the regression analysis, implies that there is something specific to each of these dimensions that has a positive effect on Objective Idiom Knowledge. Due to the specific and clear operationalizations of these dimensions, the interpretation of these positive effects is more straightforward than that of the broad operationalization of general familiarity as used by Carrol et al. (2017). Moreover, although the correlation with Objective Idiom Knowledge was low, objectively assessed idiom frequency turned out to positively affect Objective Idiom Knowledge. Adding Objective Frequency to the regression model did not change the effects of other predictors. This suggests Objective Frequency has its own unique added value in predicting idiom knowledge, albeit a medium effect only. This, together with the finding that Subjective and Objective Frequency are poorly correlated, confirms our idea that Subjective and Objective Frequency measure different aspects of frequency of exposure.

Transparency also positively influences Objective Idiom Knowledge and contributes most strongly to idiom knowledge. Transparency turned out to be especially important if participants indicated not to be familiar with the meaning of the idiomatic expression. Similarly, Libben and Titone (2008) reported an interaction effect between frequency and semantic decomposability in predicting the meaningfulness of a phrase (subjectively assessed). Here the effect of semantic decomposability was especially strong for infrequent idiomatic expressions. Although this interaction effect is slightly different from the interaction effect of Familiarity and Transparency in our study, the underlying reasoning is similar. If participants indicate they are not familiar with an idiomatic expression, they arrive at the meaning of the expression more easily if the idiom is transparent, rather than opaque. This is because in the case of a transparent idiom, the individual words can be used to arrive at the figurative meaning. If participants indicate to be familiar with the meaning of the idiomatic expression, Transparency does not affect their performance on the knowledge test, because they know the meaning anyway.

Imageability has a significant, negative impact on idiom knowledge that is stronger for familiar idiomatic expressions than for unfamiliar idiomatic expressions. The direction of the effect is in contrast with earlier studies on idiom learning (Steinel et al., 2007; Boers et al., 2008). Presenting an image of the idiom is found to enhance the link between the form and the meaning of the idiomatic expression (Steinel et al., 2007), resulting in higher learning gains. However, participants may have formed an image of the literal interpretation, rather than of the figurative meaning. This would be in line with Cacciari and Glucksberg (1995), who found that participants more often depict the literal meaning of the idiomatic expression than the figurative meaning. As a result, Imageability negatively affected idiom processing. In the current study, forming an image of the literal interpretation interferes with correctly identifying the idiom’s meaning, especially when the participants say to be familiar with the meaning. If participants are not familiar with the meaning, forming a literal image of the idiom hinders correct recognition of the meaning to a lesser extent.

Being able to form a literal image of the idiom may be related to another idiom property: literal plausibility (Libben and Titone, 2008) or Literality (Cieślicka, 2006, 2013; Beck and Weber, 2016a). This is the extent to which an idiom can be interpreted literally. Libben and Titone (2008) reported a negative effect of literal plausibility on reaction times to idiomatic expressions, an online comprehension measure. Literal plausibility and imageability might be related, because one can relatively easy form an image of idiomatic expressions that are highly literally plausible. This will probably be an image of the literal interpretation, which might interfere with idiom knowledge. For idiomatic expressions that are not literally plausible, the extent to which people are able to form an image may depend on the extent to which they know the meaning of the idiomatic expression. Only if they are familiar with the meaning of the expression, will they be able to form an image of the figurative reading of the idiom. In this latter case, the effect of Imageability would be positive.

## Conclusion

Our study addressed subjective judgments by native speakers on idiom properties that are often employed in psycholinguistic research, with the explicit aim of determining data reliability, the interrelation of the idiom properties and their impact on the participants’ idiom knowledge. To this end we performed a comprehensive rating study on Dutch idioms for which the database with idiom properties is now available (see footnote 1.).

Our reliability analysis of subjective judgements by Dutch native speakers with respect to various dimensions of Dutch idiomatic expressions leads us to recommend that future norming studies on idiomatic expressions use the D-coefficient, which is part of Generalizability Theory, as a measure of reliability. The D-coefficient can handle all kinds of research designs and measurement levels, and it allows to generalize across raters. This metric also allows to assess the minimum number of raters that are required to obtain reliable data.

Our study shows that, based on this analysis, the dimensions can be divided in two groups: experience-based dimensions (Familiarity, Subjective Frequency, and Usage), and content-based dimensions (Transparency, and Imageability). For experience-based dimensions that are carefully operationalized, 10 raters might be sufficient to obtain reliable data, whereas for judgements of the content-based dimensions to be reliable at least 20 participants are required.

Furthermore, the discrepancies between subjective and objective idiom frequency, as observed in this study, suggest that these variables measure different aspects of frequency of exposure. Additional research is necessary to clarify these discrepancies.

Moreover, we found that Transparency, Familiarity, and Imageability most strongly influenced Objective Idiom Knowledge. Imageability negatively influenced idiom knowledge. This negative effect may have been due to a lack of specificity in operationalization, because it is hard to determine whether participants formed an image of the literal or figurative interpretation.

We therefore recommend to researchers that they carefully operationalize idiom properties for their norming studies and assess whether the collected subjective judgements are reliable by using the D-coefficient.

## Ethics Statement

This study was carried out in accordance with the recommendations of the Ethics Assessment Committee (EAC) of the Faculty of Arts of Radboud University Nijmegen (number 3382) with written informed consent from all subjects. All subjects gave written informed consent in accordance with the Declaration of Helsinki. The protocol was approved by the Ethics Assessment Committee (EAC) of the Faculty of Arts of Radboud University Nijmegen.

## Author Contributions

All authors contributed to the conception and design of the study, manuscript revision, and read and approved the submitted version. FH and CC created the materials, and were involved in the data collection. FH performed the statistical analyses and organized the database. FH and CC wrote the first draft of the manuscript.

## Conflict of Interest Statement

The authors declare that the research was conducted in the absence of any commercial or financial relationships that could be construed as a potential conflict of interest.

## Footnotes

1 https://doi.org/10.17026/dans-zjx-hnsk
