# Supplementary material for: Normative Data of Dutch Idiomatic Expressions: Subjective Judgments You Can Bank on
Source: Front Psychol. 2019 May 14;10:1075. doi: 10.3389/fpsyg.2019.01075 (PMC6527779; doi:10.3389/fpsyg.2019.01075)
Supplement: Supplementary file 1 [file Data_Sheet_1.PDF]

Supplementary Material

Normative Data of Dutch Idiomatic Expressions: Subjective Judgments You Can Bank on

Ferdy Hubers*, Catia Cucchiarini, Helmer Strik, Ton Dijkstra

*** Correspondence:** Ferdy Hubers: f.hubers@let.ru.nl

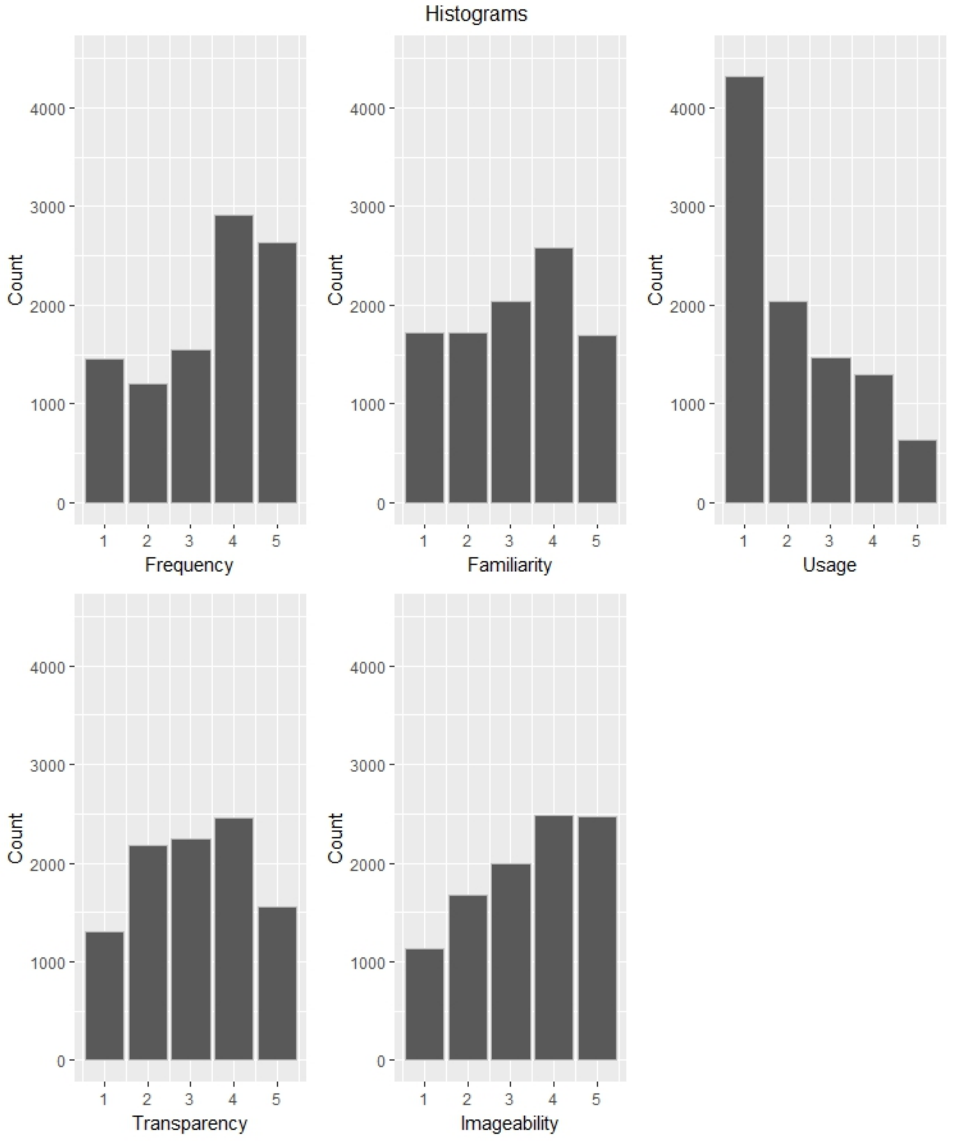

Supplementary Figure S1. Histograms based on the individual data for the idiom properties Frequency, Familiarity, Usage, Transparency, and Imageability.

Supplementary Table S1. The Reliability Coefficients per Experimental List and Dimension, and the Properties of the Experimental Lists (Number of Raters and Number of Items)

Dimension	List	D-coefficient	ICC	Cronbach's α	Krippendorff's α	#Raters	#Items
Familiarity	1	.944038	.944038	.961795	.354117	29	25
Familiarity	2	.97166	.97166	.978655	.497805	33	25
Familiarity	3	.954178	.954178	.963495	.397765	30	25
Familiarity	4	.968623	.968623	.97565	.5039	29	25
Familiarity	5	.937957	.937957	.955598	.314853	31	25
Familiarity	6	.972708	.972708	.978926	.515407	32	25
Familiarity	7	.945133	.945133	.956449	.360562	29	25
Familiarity	8	.970852	.970852	.974698	.532486	28	25
Familiarity	9	.912504	.912504	.928184	.299714	23	25
Familiarity	10	.932222	.932222	.950861	.405387	19	25
Familiarity	11	.908748	.908748	.952159	.283412	23	25
Familiarity	12	.939557	.939557	.958009	.410853	21	25
Familiarity	13	.910154	.910154	.935299	.291308	23	25
Familiarity	14	.911845	.911845	.932944	.326668	20	25
Familiarity	15	.963566	.963566	.970915	.557914	20	25
Frequency	1	.950402	.950402	.964149	.384871	29	25
Frequency	2	.965997	.965997	.975277	.450335	33	25
Frequency	3	.93831	.93831	.951938	.324294	30	25
Frequency	4	.974008	.974008	.977665	.553056	29	25
Frequency	5	.940249	.940249	.952233	.324969	31	25
Frequency	6	.975482	.975482	.979484	.543393	32	25
Frequency	7	.949203	.949203	.958	.380205	29	25
Frequency	8	.963338	.963338	.970392	.472401	28	25
Frequency	9	.921486	.921486	.942622	.32377	23	25
Frequency	10	.941838	.941838	.956475	.446472	19	25
Frequency	11	.917354	.917354	.944608	.310111	23	25
Frequency	12	.929018	.929018	.95007	.369147	21	25
Frequency	13	.927015	.927015	.941395	.343109	23	25
Frequency	14	.894189	.894189	.924468	.281383	20	25

Frequency	15	.957923	.957923	.966401	.520365	20	25
Usage	1	.949582	.949582	.961933	.381149	29	25
Usage	2	.964247	.964247	.975731	.436913	33	25
Usage	3	.926376	.926376	.949603	.281884	30	25
Usage	4	.964378	.964378	.975027	.470134	29	25
Usage	5	.955116	.955116	.965766	.394754	31	25
Usage	6	.966279	.966279	.974595	.460375	32	25
Usage	7	.954547	.954547	.964407	.407829	29	25
Usage	8	.952831	.952831	.964899	.406339	28	25
Usage	9	.880791	.880791	.914311	.228439	23	25
Usage	10	.936056	.936056	.947211	.422589	19	25
Usage	11	.86544	.86544	.918886	.20054	23	25
Usage	12	.896312	.896312	.928875	.275593	21	25
Usage	13	.941163	.941163	.955435	.39694	23	25
Usage	14	.888255	.888255	.929712	.26639	20	25
Usage	15	.944858	.944858	.958609	.448007	20	25
Transparency	1	.908099	.908099	.93474	.240857	29	25
Transparency	2	.841655	.841655	.888866	.126672	33	25
Transparency	3	.893311	.893311	.927459	.204606	30	25
Transparency	4	.800794	.800794	.860116	.108559	29	25
Transparency	5	.895209	.895209	.917063	.204987	31	25
Transparency	6	.911889	.911889	.946516	.22982	32	25
Transparency	7	.872751	.872751	.910779	.177956	29	25
Transparency	8	.865019	.865019	.893447	.174876	28	25
Transparency	9	.891645	.891645	.911894	.251216	23	25
Transparency	10	.897114	.897114	.923722	.299384	19	25
Transparency	11	.770516	.770516	.834104	.112292	23	25
Transparency	12	.838986	.838986	.894329	.181034	21	25
Transparency	13	.879923	.879923	.924142	.224647	23	25
Transparency	14	.824009	.824009	.863578	.175373	20	25
Transparency	15	.9049	.9049	.941348	.304621	20	25
Imageability	1	.932088	.932088	.947944	.308823	29	25
Imageability	2	.907145	.907145	.919982	.218855	33	25

Imageability	3	.909603	.909603	.930803	.239169	30	25
Imageability	4	.862856	.862856	.895845	.166431	29	25
Imageability	5	.904102	.904102	.915526	.223782	31	25
Imageability	6	.889492	.889492	.906145	.191449	32	25
Imageability	7	.934169	.934169	.94646	.316874	29	25
Imageability	8	.922048	.922358	.94038	.292898	27	25
Imageability	9	.841507	.841507	.891227	.171691	23	25
Imageability	10	.847942	.847942	.872964	.214334	19	25
Imageability	11	.737634	.737634	.820427	.092123	23	25
Imageability	12	.825518	.825518	.872602	.168423	21	25
Imageability	13	.898165	.898165	.922728	.263537	23	25
Imageability	14	.810732	.810732	.857463	.161194	20	25
Imageability	15	.931114	.931114	.947706	.389455	20	25

Supplementary Table S2. Variance Inflation Factors (VIFs) for the final regression analysis

**Fixed effects**	**VIF**
Familiarity	2.31
Transparency	1.14
Imageability	1.62
Frequency	1.90
Usage	1.49
Objective idiom frequency	1.02
Familiarity x Transparency	1.37
Familiarity x Imageability	1.56
